# PASS2 database for the structure-based sequence alignment of distantly related SCOP domain superfamilies: update to version 5 and added features

**DOI:** 10.1093/nar/gkv1205

**Published:** 2015-11-08

**Authors:** Arumugam Gandhimathi, Pritha Ghosh, Sridhar Hariharaputran, Oommen K. Mathew, R. Sowdhamini

**Affiliations:** 1 National Centre for Biological Sciences (TIFR), GKVK Campus, Bangalore 560065, Karnataka, India; 2 Bharathidasan University, Palkalainagar, Tiruchirapalli 620024, Tamilnadu, India; 3 SASTRA University, Tirumalaisamudram, Thanjavur 613401, Tamil Nadu, India

## Abstract

Structure-based sequence alignment is an essential step in assessing and analysing the relationship of distantly related proteins. PASS2 is a database that records such alignments for protein domain superfamilies and has been constantly updated periodically. This update of the PASS2 version, named as PASS2.5, directly corresponds to the SCOPe 2.04 release. All SCOPe structural domains that share less than 40% sequence identity, as defined by the ASTRAL compendium of protein structures, are included. The current version includes 1977 superfamilies and has been assembled utilizing the structure-based sequence alignment protocol. Such an alignment is obtained initially through MATT, followed by a refinement through the COMPARER program. The JOY program has been used for structural annotations of such alignments. In this update, we have automated the protocol and focused on inclusion of new features such as mapping of GO terms, absolutely conserved residues among the domains in a superfamily and inclusion of PDBs, that are absent in SCOPe 2.04, using the HMM profiles from the alignments of the superfamily members and are provided as a separate list. We have also implemented a more user-friendly manner of data presentation and options for downloading more features. PASS2.5 version is available at http://caps.ncbs.res.in/pass2/.

## INTRODUCTION

The determination of structural relationships between proteins is fundamental in biological science to classify proteins, to analyze and predict protein function, or to support the prediction of experimentally yet undetermined protein structures (1). Protein structure alignment methods are important in understanding the structural, evolutionary and functional relationships between proteins. It is even more challenging to perform alignments for distantly related proteins owing to their high sequence divergence. Computation of structure-based alignment, that either employ rigid-body superposition methods or local environment of residues or both can give rise to more reliable alignments for distant relationships, when compared to pure sequence alignments (2).

Protein domains grouped together at the superfamily level are defined as having structural, functional and sequence similarities and evidence for a common evolutionary ancestor. Structure-based sequence alignments of distantly related proteins are rarely studied, but could serve as reliable evolutionary models. PASS2 (3), provides structure-based alignments for domains within superfamilies and is in accordance with the Structural Classification of Proteins (SCOP) database since 1998 (4). The SCOPe database (5) is an extended version of the SCOP database, and employs automated methods combined with manual curation to classify newer structures. The authors of the SCOPe database claim that its accuracy matches the hand-curated SCOP releases. Protein structural domains, which are no more than 40% identical to each other in sequence within a superfamily, were chosen from SCOP database for alignment and inclusion into PASS2 database. This filter was useful in order to reduce the computational time of applying rigorous structure comparison methods on closely related structural entries, where simple sequence alignments are relatively straightforward.

Many databases have been developed for understanding of structure-function relationships of proteins related at family and/or superfamily level. Few pertinent databases are alone mentioned here, out of large number of examples. The HOMSTRAD (6) database contains aligned 3D structures of homologous proteins. SUPFAM database (7) deals with protein superfamily relationships derived by comparing sequence-based and structure-based families. The DALI database (8) contains all-against-all structure comparison of protein structures in the Protein Data Bank (PDB) and retain automatically maintained and regularly updated structural alignments. PALI (9) is another database providing Phylogeny and ALIgnment of homologous protein structures and contains structure-based sequence alignments. VAST (Vector Alignment Search Tool) is an algorithm to identify protein three-dimensional structural similarities based on purely geometric criteria and is applicable for homologues that are distant from each other in sequence space (10). The PASS2 database is unique in dealing with alignments of distantly related protein domain superfamilies and has been consistently updated with improvements along with SCOP versions (11–14).

We present an update of the PASS2 database, namely PASS2.5, which directly corresponds to the SCOPe 2.04 release. This update of PASS2 involves a greater number of structures recorded in the SCOPe database, an improved protocol with additional features such that the approach is robust to handle diverse types of superfamilies.

## ALIGNMENT PROTOCOL

The structural domains have been obtained from ASTRAL 2.04 which corresponds to the SCOPe 2.04 version. The superfamilies were further classified based on their number of domains and accordingly names as single-member superfamilies (SMS) and multi-member superfamilies (MMS). In this update, the two-member superfamilies (TMS) were also considered with MMS owing to similar nature of tools and methods employed for both the sets. An initial alignment and superposition was performed using MATT (15) program. From the initial alignment, equivalent regions were identified (non-gapped aligned regions) and retrieved using the JOY-4.0v program (16). These initial equivalences and the structure-guided tree information are the typical inputs for COMPARER (17) program. COMPARER alignment procedure employs variable gap penalties and local structural features such as backbone conformation, solvent accessibility and hydrogen bonding patterns. In general, the variable gap penalties ensure that there are no unreasonable gaps in between secondary structures and conserved regions within the alignment. After the final alignment through COMPARER, JOY-3.2v program is employed to recognize all non-gap alignment positions as equivalences. Such equivalences are employed for rigid-body superposition using MNYFIT (18) to obtain superimposed structures, through Euclidean transformations with no further modification to the equivalences.

### Implementation and data organization

In this version, MySQL 5.2 was employed as database engine while Python2.7 and BioPython (19) has been used for implementing the back-end data retrieval and manipulation logic. The user interface has been built on components from HTML5, CSS, JavaScript, Ajax and JQuery. The visualization of the molecular structures and phylogenetic tree has been implemented using JSMol and raphael and jsPhyloSVG (20), while the visualization of the alignment and mapping of conserved residues have been implemented using in-house plug-in.

## FEATURES

**Figure 1.**
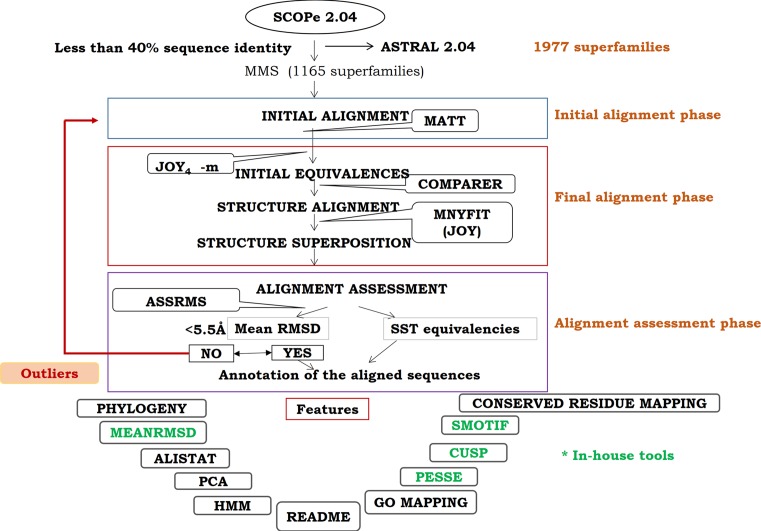
Flow-chart for rigorous structure-based sequence alignment of distantly related proteins. The flow-chart describes three phases namely initial alignment phase, final alignment phase and alignment assessment phase. The features are mentioned at the end (outside rectangular boxes). In particular, features marked in green are in-house developed programs for annotation.

As in the previous versions, each PASS2 superfamily is provided with the information such as HMM (21,22), structural motifs (using SMotif) (23), structural phylogeny, PCA analysis and indel regions (using CUSP) (24). At the domain level, the accessory files such as PSA, HBD and SST provided by the JOY program are also available for download. For each superfamily in the PASS2 database, HMM profile is constructed by employing ‘hmmbuild’ from HMMER suite. The sequence similarity distribution of members of a superfamily was shown in a 3D projection/plot PCA plots (25). The SMotif program is employed to identify structural motifs from an aligned set of protein structures on the basis of conservation of amino acid preferences and solvent inaccessibility. These are then examined for conservation of other features like secondary structural content, hydrogen bonding and residue packing. The CUSP algorithm identifies indels by examining protein domain structural alignments to distinguish ‘core’ structural regions that are conserved among related proteins from regions that vary in length and type of structure. Alistat provides information about basic statistics about the superfamily alignment such as the number of sequences, the total number of residues, the average length of sequences and the range of sequence lengths, the alignment length (including gap characters) and sequence identity information. Percentage of Conserved Secondary Structural Equivalences (COSSE) and the mean RMS on values are calculated for superfamily members. The structural phylogeny was constructed using RMSD matrix, which was derived from the structural superposition of proteins within a superfamily (Figure 1).

## IMPROVEMENTS IN THE UPDATED VERSION AND APPLICATIONS

### Assigning new structural entries to pre-existing superfamilies

Previous work on recognition of distantly related proteins has shown that profiles generated from protein families of known structure, when used as start points for sensitive search methods, lead to high confidence structure associations (26). Thus, accumulation of known protein structures that lack a SCOP classification, by profile-based search methods may help assign functions based on superfamily-specific GO terms to them and also bridge the gap between the increasing number of solved structures and SCOP classification. In PASS2.5, we provide connections to more structural entries for each superfamily using the superfamily HMM profile. Each superfamily HMM had been searched against the PDB to gather more entries to the superfamily of interest.

### Mapping gene ontology terms

The gene ontology (GO) represents properties of gene product under three major terms, namely cellular component, molecular function and biological process (27). In this update, we have included GO mapping as one of the new features for each superfamily. Each superfamily members are assigned with GO term(s), which were retrieved dynamically from www.rcsb.org using the RestFul API clients written in Python and indicates the most probable functions associated with the protein chains and/or domains in a superfamily.

### Mapping absolutely conserved residues

**Figure 2.**
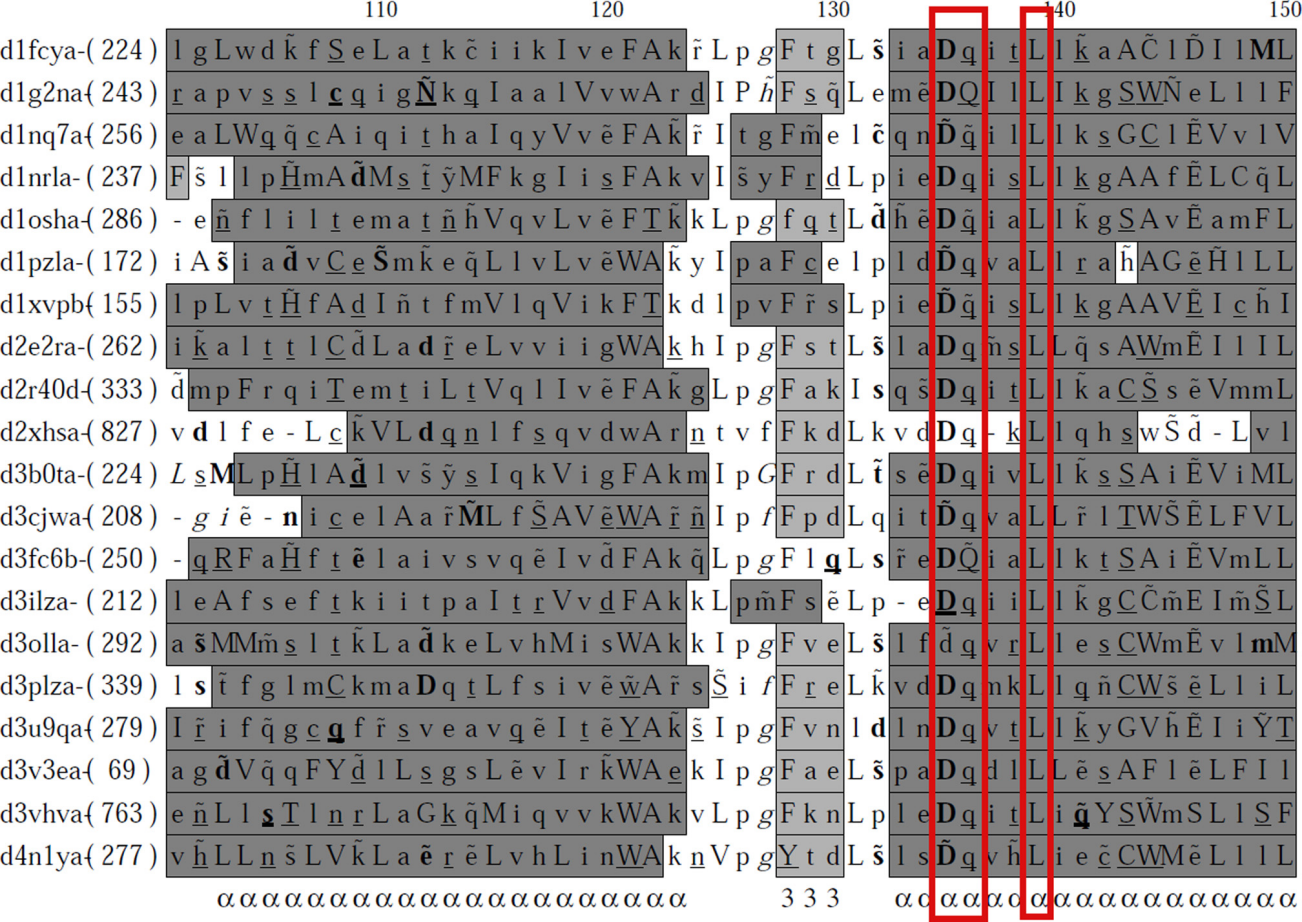
Conserved residues are mapped on part of the alignment of Nuclear receptor ligand-binding domain superfamily (SCOPe code: 48508). In the alignment, amino acids mentioned in uppercase are solvent inaccessible and residues in lowercase are solvent accessible. Hydrogen bond to main chain amide are denoted in bold,residues having hydrogen bond to mainchain carbonyl are underlined and hydrogen bond to other sidechain is indicated by a tilde (∼) over the amino acid concerned. By default, underneath the alignment is the consensus secondary structure. The definition of 'consensus’ is that a fraction of >0.7 is in a particular conformational state at a given position.

Superfamily members generally share conserved motif(s) that are important for structure and/or function. Families belonging to the same superfamily often have additional motif(s) and/or distinct residue patterns within motifs that are involved in substrate specificity or family-specific biological functions. Mapping of absolutely conserved residues onto the superfamily alignments provide clues to expand structural and functional studies on specific superfamilies where such family-specific functional outliers are prominent. In this update, we have mapped absolutely conserved residues (ACR), which are 100% identical in all the domains considered in that superfamily. ACRs are rapid pointers of important regions of a protein superfamily since a majority of FCRs might correspond to functional residues and ACRs might form a subset of functionally important residues as well. The superfamilies which have more than four members have alone been considered for such mapping. Interestingly, it was observed that the nuclear receptor ligand-binding domain superfamily, having 20 members, show 100% conservation for two polar residues—aspartate and glutamine and a hydrophobic residue Leucine (Figure 2). These three ACRs are located on the third helix closer to the N-terminus and could be important for structural integrity. Such information on ACRs, in general, may be useful to identify the superfamily signature residues or functionally important residues.

### Structurally deviant domains of the superfamily

Alignment of protein structures are generally measured by RMSD which provides a measure of the average distance between aligned C^α^ atoms of superimposed proteins. There is an increasing evidence in some superfamilies of domains that have undergone significant structural changes during evolution (28,29). Such superfamilies with members of high conformational variability will pose a challenge for any structural alignment program. This approach of looking at protein structure alignments at a superfamily level have provided us with a vast understanding of the similarities and deviations among the members pointing towards their subtle differences in functions.

The suggested protocol provides good alignment accuracy with low RMSD. It still permits us to identify structurally deviant members of the superfamily which we refer as outliers (Supplementary Figure S1). As in the previous update, outliers are identified and characterized from MMS (30). Out of the 1165 MMS, 243 have one or more structurally deviant member(s). It was observed that 71 superfamilies retain family-specific outliers, which means that they belong to a different family in comparison to the other members in that superfamily. The outlier(s) for a superfamily (if any) are provided as a separate file in the web interface.

## APPLICATION IN ENHANCED SEQUENCE SEARCHES: CASE STUDY WITH RRM DOMAINS

**Figure 3.**
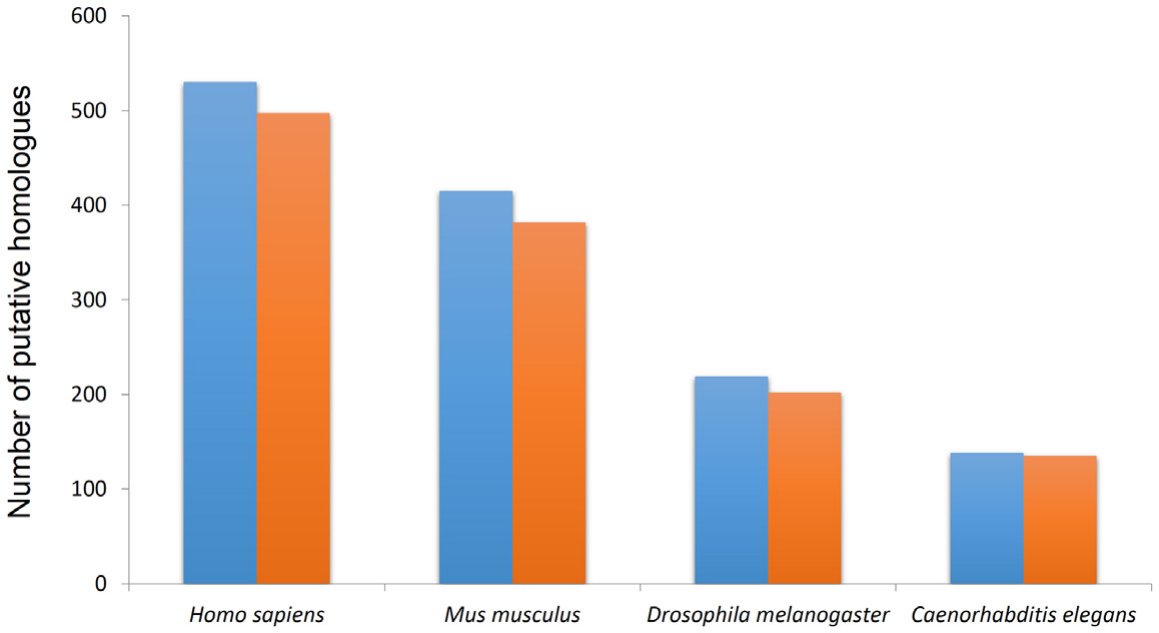
Whole-genome search for putative homologues in proteomes of four different model organisms. Results obtained by search using PASS2 HMM is shown in blue colour and search using HMM derived from the most populated RRM-1 Pfam family is shown in red. Higher coverage is obtained when searched using HMMs of PASS2 superfamilies in all the proteomes under study.

We have compared the PASS2 alignments (HMMs) with those available from other sequence domain family databases like Pfam (31). PASS2 deals with distantly related members that diversify into multiple Pfam families which include more closely related and reliable set of homologues. Hence, it is more challenging to generate alignments from PASS2 superfamilies as compared to that of Pfam families. One such example is the RNA-binding domain, RBD superfamily (SCOP id: 54928) that has diverged into at least seven Pfam RNA recognition motif families (RRM_1, RRM_2, RRM_3, RRM_5, RRM_6, RRM_7 and RRM). The length of the PASS2 HMM arising out of the RBD superfamily is almost double that of each of the Pfam families. We have compared the performance of the HMMs generated out of the RBD superfamily with that of the most populated Pfam RNA recognition motif family (RRM_1; Pfam id: PF00076) in terms of sequence search coverage in four different model organism proteomes and the results are shown in Figure 3. In all the cases, the number of putative homologues identified by PASS2 HMM-based sequence searches are more than that by Pfam HMM.

## CONCLUSION

PASS2 database provides structure-based sequence alignments of protein domain superfamilies in correspondence with SCOP definitions. The codes have now been organized in Linux platform for convenient updates in future and our alignment protocol employs improved methods of alignment. Multiple features such as CUSP, HMM, structural phylogeny, PCA and MEANRMS provide in-depth analysis of each superfamily. Superfamily descriptors were identified based on their structural motifs. HMMs of PASS2 superfamily members are useful in detecting distant relationships at poor sequence identities. New features such as mapping of GO terms, absolutely conserved residues and inclusion of new PDBs to the superfamilies are the highlights in PASS2.5 which is the current updated form of the database (Supplementary Figure S2). We have also proposed that structurally deviant superfamily members could be recognised as outliers to gauge the quality of the alignment. Structure-based sequence alignments serve as evolutionary models of distant relationships retaining similar structural properties and therefore can also enable systemic fold prediction.
